# Seasonal variation and severity of Diabetic Ketoacidosis in patients at a tertiary care hospital in Pakistan

**DOI:** 10.12669/pjms.38.5.5227

**Published:** 2022

**Authors:** Bakht Babar, Azizul Hasan Aamir

**Affiliations:** 1 Dr. Bakht Babar, FCPS Medicine, PGR, Department of Diabetes, Endocrinology and Metabolic Diseases, MTI Hayatabad Medical Complex, Peshawar, Pakistan; 2 Dr. Azizul Hasan Aamir (MRCP (UK), FRCP (Edin), FACE (US). HOD/Professor, Department of Diabetes, Endocrinology and Metabolic Diseases, MTI Hayatabad Medical Complex, Peshawar, Pakistan

**Keywords:** Seasonal variation, Severity, Diabetic Ketoacidosis (DKA), T1DM, T2DM, Pakistan

## Abstract

**Objectives:**

To asses the seasonal variability in the hospital admissions of Diabetic Ketoacidosis (DKA) patients.

**Methods:**

This two year retrospective analysis was carried out from first November 2018 to 31^st^ OCTOBER 2020, which involved medical chart reviewing of all those patients admitted to the Department of Diabetes, Endocrinology and Metabolic diseases, Hayatabad Medical complex, Peshawar, Pakistan with confirmed DKA. Data related to patient demography, past history, biochemical profile and treatment was collected and analysed using SPSS version 25.

**Results:**

During the above mentioned 24 months, 104 diabetic patients with confirmed DKA were admitted. Fifty-nine (57%) patients were male. Most of the patients had moderate DKA that is 42(40%). Seasonality was observed with majority of the patient admitted in winter season overall 62(60%). In both the years encounter with DKA patient peaked in January. DKA was found to be more severe in female and in age group 10-15 years. DKA severity was found to be significantly associated with age, gender, previous episodes of DKA, length of hospital stay and non compliance plus infection (p<0.05).

**Conclusion:**

We found seasonal variation and peaked cases of DKA in the winter season presenting at a tertiary care hospital in Pakistan. Noncompliance was found to be major contributory factor.

## INTRODUCTION

Diabetic ketoacidosis (DKA) is one of the most common life-threatening emergency which occurs in decompensated diabetes (both Type-I and Type-II DM). DKA results from absolute or relative insulin deficiency which leads to hyperglycemia and accumulation of ketone bodies in the blood as a substitute substrate for energy, with subsequent metabolic acidosis.^1^ It is one of the major cause of mortality and morbidity in diabetes.^2^,^3^ Most of the DKA related death occur due to development of cerebral edema.^4^ In United States the incidence of DKA per 10,000 admission rose from 32.04 in 2003 to 61.6 in 2017.^5^ Local studies done in Pakistan showed the frequency of DKA to be 2.2% in children with previously known diabetes and 6.7% with newly diagnosed diabetes.^6^

Seasonality is a very common phenomena that is seen in many health-related problems. Seasonal variability of certain diseases like influenza and respiratory syncytial virus is quite common, however now evidence is emerging with proved seasonality in hospitalization for other conditions like asthma, congestive cardiac failure and myocardial infarction.^7^-^9^ Similarly, there are strong evidences for seasonal variation of DKA as shown by many international studies.^10^-^14^

Pakistan lies in the temperate zone. Its inhabitant enjoys four seasons per year i.e. Winter (Mid November to February), Spring (March and April), Summer (May to September), and Autumn (October to Mid November), as confirmed from Pakistan Meteorological department. This study looked at the seasonal variation and its effect on the number of patients admitted for DKA to the department of diabetes, endocrinology and metabolic diseases, Hayatabad medical complex Peshawar. Peshawar is the capital of Pakistani province Khyber Pakhtunkhwa, located at a latitude of 34.008 and longitude of 71.57849 and is 3781.1km from the equator lying in the northern hemisphere. In addition we also looked at the association of various clinical and biochemical parameter with degree of severity of DKA.

DKA is a very neglected health issue in Pakistan. Currently there is no data regarding seasonal variability of DKA in Pakistani population, likewise for severity of DKA very limited literature can be found. So, this study was of immense importance to fill this gap, and will help the physician and health care administrator to better anticipate and prepare for seasonal fluctuation of DKA, by making all the necessary arrangement to cope with it and make all supplies available in a resource constraint settings to minimize the loss of life. Result may also help to educate people about time when they are more vulnerable. Most importantly it will be a gateway for further research work, identifying underlying causes so that appropriate preventive measures that encompass a variety of interventions, which can be undertaken.

## METHODS

This was a two year retrospective study which involved reviewing medical charts of all those diabetic patients who had confirmed DKA, and admitted to Endocrinology Department, Hayatabad medical complex, Peshawar, KPK, during the time period from 1^st^ November 2018 to 31^st^ October 2020 after approval from ethical committee of the hospital (Refence no. 382/HEC/B&PSC/2020, Dated 14 January 2021). The diagnosis of DKA was based on the characteristics finding, including elevated serum ketones, Blood glucose >250mg/dl, Ph <7.3 and HCO3 <15 mmol/l in established diabetes mellitus as per ADA criteria.**1** DKA has been classified into three categories on the basis of severity. This classification has been done on the basis of Ph, HCO3 and anion gap. So, Severity of DKA was defined as Mild (ph7.24-7.30, plus HCO3 15- 18mmol/l, and anion gap>10), Moderate (Ph7.0-7.24, plus HCO3 10- 15mmol/l, and anion gap>12), and Severe (Ph<7.0, plus HCO3 <10mmol/l, and anion gap>12), as per ADA criteria.^1^

All the patient data regarding demographic details, past history related to diabetes and its complication, biochemical parameters at presentation, information related to precipitating factors, severity of DKA, details regarding treatment including amount of insulin infused, time for resolution of DKA, length of hospital stay and outcome were recorded on a well, designed questionnaire.

Statistical analysis was performed using SPSS ver.25.0. The qualitative data of patients were analyzed and expressed as frequency and percentages. The quantitative data was analysed and expressed as (means ± standard deviations). For analysing the differences in participants’ characteristics by severity of ketoacidosis category, ANOVA and χ2 test were used for analysis of variance for continuous data and categorical data, respectively. Statistical significance was defined as P < 0.05 for all data.

## RESULTS

Data of 104 patients was analyzed for this study. Patients were classified into three categories on the basis of mode of severity that is mild (n=30), moderate (n=42) and severe DKA (n=32). Majority of patient i.e 40% were in moderate category. Out of 104 patients 59 (57%) were male and 45 (43%) were female. A significant association was found between gender and Severity, and female mostly presented with severe DKA (p= 0.025). DKA presentation was highest among age group >20 years i.e 32 (31%) closely followed by age group 10 – 15 years who also recorded highest rate of severe DKA as compared to other age categories (p=0.002). DKA was found to be highest in those who were known Type-I diabetic and also in those who were known diabetic for the past 1 - 5 years and no significant association was found with various categories of severity (p>0.05). Previous episode of DKA was found to be significantly associated with severity of DKA and those who had previously 1-4 episodes were the one effected the most (p=0.02). Noncompliance was the commonest precipitating factor and had the greatest impact and was responsible for DKA in more than fifty percent patient i.e 56 (54%) (p=0.002). (Table-I)

**Table I:** Baseline characteristics of various modes of severity of DKA.

	Mild n (%)	Moderate n (%)	Severe n (%)	Total n (%)	P Value (significant only)
Counts	30 (29)	42 (40)	32 (31)	104	
**Gender**					0.025
Male	23 (39)	22 (37)	14 (24)	59 (57)	
Female	7 (16)	20 (44)	18 (40)	45 (43)	
**Age (Years)**					0.002
0 – 5 years	0 (-)	1 (50)	1 (50)	2 (2)	
6 – 10 years	10 (40)	15 (60)	0 (-)	25 (24)	
10 – 15 years	7 (23)	6 (20)	17 (57)	30 (29)	
15 – 20 years	6 (40)	4 (27)	5 (33)	15 (14)	
>20 years	7 (22)	16 (50)	9 (28)	32 (31)	
**Season of Admission**					0.513
Winter	17 (27)	26 (42)	19 (31)	62 ((60)	
Spring	3 (33.3)	3 (33.3)	3 (33.3)	9 (9)	
Summer	10(38.5)	10 (38.5)	6 (23)	26 (25)	
Autumn	0 (-)	3 (43)	4 (57)	7 (6)	
**Type of DM**					0.509
Newly diagnosed Type-I DM presented with DKA	4 (25)	7 (44)	5 (31)	16 (15)	
Known Type-I DM presented with DKA	24 (33)	29 (40)	20 (27)	73(70)	
Known Type-II DM presented with DKA	2 (13)	6 (40)	7 (47)	15 (15)	
**Duration of Diabetes (Years)**					0.973
Newly diagnosed	4 (25)	7 (44)	5 (31)	16 (15)	
Less than a year	3 (27)	6 (55)	2 (18)	11 (11)	
1 – 5 years	13 (33.3)	13 (33.3)	13 (33.3)	39 (37)	
6 – 10 years	7 (27)	11 (42)	8 (31)	26 (25)	
>10 years	3 (25)	5 (42)	4 (33)	12 (12)	
**Comorbidities** (IHD, DKD, HTN etc.)*					0.511
Yes	2 (15)	6 (46)	5 (39)	13 (12.5)	
No	28 (31)	36 (40)	27 (29)	91 (87.5)	
**Previous Episodes of DKA**					0.02
No Episode	4 (15)	15 (58)	7 (27)	26 (29)	
1 – 4 Episodes	19 (44)	13 (30)	11 (26)	43 (49)	
>4 Episodes	3 (16)	7 (37)	9 (47)	19 (22)	
**Precipitating Factors**					0.002
Non Compliance	22 (39)	25 (45)	9 (16)	56 (54)	
Infection	5 (28)	6 (33)	7 (39)	18 (17)	
Both Noncompliance and infection	2 (8)	7 (29)	15 (63)	24 (23)	
Other causes	1 (17)	4 (66)	1 (17)	6 (6)	

* IHD (Ischemic Heart Disease), DKD (Diabetic Kidney Disease), HTN (Hypertension).

**Fig.1:**
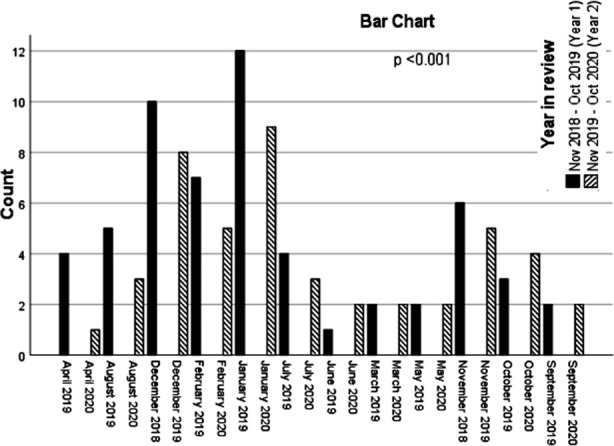
Month and year of Admission.

**Fig.2:**
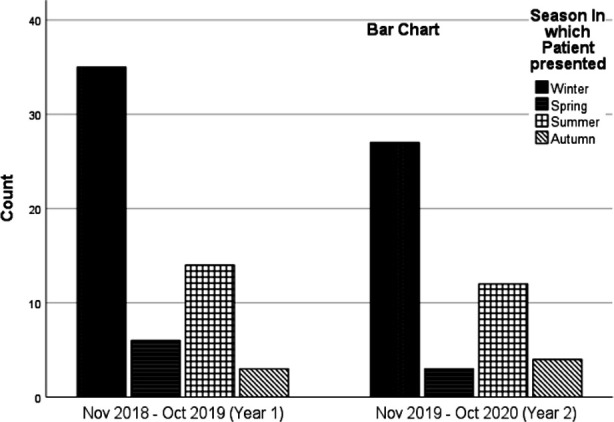
Year wise distribution of DKA in different seasons.

The bar graph (Fig.1) illustrate seasonal trends and month wise presentation of DKA. DKA presentation in different season follow almost similar trend in both the years with highest number of cases recorded in winter and lowest in Autumn in year one and spring in year two. DKA patients peaked in January in both the years. (Fig.2)

Table-II compares the biochemical parameters of various mode of severity of DKA. Primary parameters for DKA diagnosis i.e. Ph, HCO3, PCO2, O2 Sat, serum ketones and anion gap was found to be significantly associated with DKA severity (p<0.001). Lab investigations like white cell count, platelets count, blood urea were also found to be higher in severe DKA as compared to the other two categories (p<0.05). No significant difference was found in other biochemical parameters.

**Table II:** Biochemical parameters of various modes of severity of DKA.

	Mild (Mean±SD)	Moderate (Mean±SD)	Severe (Mean±SD)	Total (Mean±SD)	P Value (Significant only)
Arterial Ph	7.3±0.02	7.2±0.05	6.8±0.15	7.1±0.22	<0.001
Bicarbonate (mmol/l)	15.4±0.5	12.4±1	7.3±1.7	11.7±3.4	<0.001
PCO2 (mmHg)	28.6±4.4	27.5±5.3	20±4.5	25±6.1	<0.001
O2 Saturation (%)	95±2	96±2	94±2	95±2	0.001
Serum Ketones	3.5±0.7	4.3±1.0	6.1±1.1	4.6±1.4	<0.001
Sodium (mmol/l)	135±4	134±5	133±6	134±5	0.253
Potassium (mmol/l)	4.2±0.5	4.3±0.6	4.4±0.6	4.3±0.6	0.674
Chloride (mmol/l)	100±6	98±5	101±8	99±7	0.093
Random Blood Sugar (mg/dl)	4446±106	467±92	504±95	472±99	0.064
HbA1c %	11.2±2	11.4±1.4	12±2	11.5±2	0.283
Hemoglobin (gm/dl)	12.6±1.2	12±2	11.7±2.2	12±2	0.114
WBC Count (×10E9/L)	13.6±2.1	11±5.2	18.03±9.2	14±1.3	0.064
Platelet Count (×10E9/L)	244.3±148	245.4±131.2	330±162	271±150.2	0.025
S. Creatinine (mg/dl)	0.6±0.3	0.8±0.7	1.0±0.8	0.8±0.6	0.157
BUN (mg/dl)	39±17.3	49±30	67±21	52±26.3	<0.001
Anion Gap					<0.001
10 – 12	12 (100%)	0 (-)	0(-)	12 (11.5%)	
>12	18 (20%)	42 (46%)	32 (34%)	92 (88.5%)	

Severe DKA showed statistically significant difference from mild to moderate categories in terms of time required for correction, amount of insulin used for the correction, and admission to ICU(p<0.001) (Table-III). Criteria for ICU admission was hemodynamic instability, Respiratory insufficiency, Altered mental status (Low GCS) and Severe Acidosis. Most of the patients i.e 62 (60%) stayed in hospital for 1 - 3 days and most of these were in moderate category i.e 28 (45%) followed by mild category 25 (40%), while the patient in severe category stayed mostly for 4-6 days. Some of the patient in mild and moderate category stayed in the hospital for 4-6, or more than six days, because although their DKA was resolved but they still had ongoing infection that needed to be treated fully plus they needed further diabetic education. The total duration of hospital stay had a significant association with degree of severity of DKA (p<0.001).

**Table III:** Modes of severity of DKA vs treatment outcome.

	Mild Mean ±SD n (%)	Moderate Mean ±SD n (%)	Severe Mean ±SD n (%)	Total Mean ±SD n (%)	P Value (Significant only
Time required for correction of DKA (Hours)	7.7±3	12±8.2	21±10	13.4±9.3	<0.001
Amount of insulin (Units) used for correction	35±18	60±49	88±48	62±46.4	<0.001
Admission in ICU					<0.001
No	30 (32%)	41 (43%)	24 (25%)	95 (91%)	
Yes	0 (%-)	1 (11%)	8 (89%)	9 (9%)	
Duration of hospital stay (Days)					<0.001
1 – 3 Days	25 (40%)	28 (45%)	9 (15%)	62 (60%)	
4 – 6 Days	4 (11%)	12 (33%)	20 (56%)	36 (35%)	
>6 Days	1 (17%)	2 (33%)	3 (50%)	6 (5%)	
Outcome of treatment					0.321
Recovered and discharged	30(29%)	42(41%)	31(30%)	103(99%)	
Died	0	0	1(100%)	1(1.0%)	

## DISCUSSION

Finding of our study revealed DKA to be more common in winter as compare to other seasons. Detail review of the literature showed seasonal variability of glycemic control, for example in one of Chinese study it was found that Hba1c was higher in winter as compared to summer.^10^ Butalia et al. showed temporal variation in hospitalization for DKA, which was in- agreement with Korean study Lee et al.^11^,^12^ Our findings are consistent with many international studies.^10^-^12^ However there are some studies for example Al sheikh et al. from Saudi Arabia and an Iranian study Razavi et al. which reported DKA to be more common in summer.^13^,^14^ The major reason for this difference from Iran and Saudi Arabia is the long, scorchy summer compared to our study population. On the contrary healthcare providers in this part of the world need to be on the high alert during the particular risky months of winter in to provide uninterrupted health care.

Our study demonstrated significant association of age and gender with degree of severity of DKA. DKA was found to be more common in male and in age group more than 20. However, for severe DKA we found preponderance of women that is in-agreement with other studies.^14^,^15^ Our study also reported severe DKA to be predominant among age group 15 -20, which is not supported by other studies showing no association of age with severity of DKA.^16^

Results of our study suggested that the average length of stay of a DKA patients for the most part was 1-3 days, a finding that is compatible with many studies like Desai et al. and Zhong et al both of which were UK based studies.^17^,^18^ Also DKA was found to be more common in Type-I diabetic in our study and is a universal finding in almost all related studies. However, our study reported that type of diabetes had no significant association with mode of severity of DKA.

Noncompliance was reported as one of the most common precipitating factor for DKA.^17^,^19^,^20^ Results of our study substantiated this finding and found non compliance as the most common cause for DKA. Most of these patients were diagnosed in the periphery hospitals where unfortunately education system regarding diabetes is not well developed compared to our tertiary hospital where we have properly trained staff for that. So these patients were not very well educated as they should be. This gap in health service is an eye opener for the healthcare provider to look for its causes which to our understanding are mostly due to low literacy rate, lack of education and growing cost of insulin. From public health perspective we believe that this problem can be addressed by creating different programs at the Government level and mass campaign should be started to not only educate patients and their parents, but in fact the whole community.

This research work might help the healthcare worker to better educate the diabetic about the time when they are more at risk for DKA. Moreover, noncompliance was found to be a major problem which again reinforce the idea of educating patients. Lastly, more research work need to be carried out in order to look deep into the factors causing seasonal variation.

### Strength and Limitations of the study:

This study had some limitations firstly as it was a single center study which involved retrospective medical records review. Also, we had to exclude about four medical records because of incomplete data. Despite these limitations the sample size that is the number of DKA patient is one of the highest recorded number presented to any other hospital in KPK. Secondly, no study is conducted in Pakistan on this topic and hence this is a novel study, so this will provide a solid ground for more future prospective studies.

## CONCLUSION

This study showed seasonal variation of DKA in our region with peak in the winter season. Females were most commonly affected with severe disease. Severity of the DKA was related to longer hospital stay.

### Authors’ Contribution:

**BB:** Principal investigator, Data collection and compilation, Manuscript writing, statistical analysis, Literature search, Accuracy of the work. **AHA**: Proposed the idea, Supervision, Reviewed the manuscript, Final approval.
